# Antioxidant Activity and Microbial Quality of Freeze-Dried, Lactic Acid Fermented Peach Products

**DOI:** 10.3390/molecules30112360

**Published:** 2025-05-29

**Authors:** Szymon Ossowski, Katarzyna Rybak, Katarzyna Pobiega, Joanna Sękul, Zuzanna Domżalska, Klaudia Gregorek, Anna Gramza-Michałowska, Emilia Janiszewska-Turak

**Affiliations:** 1 Department of Food Engineering and Process Management, Institute of Food Sciences, Warsaw University of Life Sciences—SGGW, 159C Nowoursynowska St., 02-787 Warsaw, Poland; s206700@sggw.edu.pl (S.O.); katarzyna_rybak@sggw.edu.pl (K.R.); s208829@sggw.edu.pl (Z.D.); s208837@sggw.edu.pl (K.G.); 2 Department of Food Biotechnology and Microbiology, Institute of Food Sciences, Warsaw University of Life Sciences—SGGW, 159C Nowoursynowska St., 02-787 Warsaw, Poland; katarzyna_pobiega@sggw.edu.pl (K.P.); s212132@sggw.edu.pl (J.S.); 3 Department of Gastronomy Science and Functional Foods, Faculty of Food Science and Nutrition, Poznan University of Life Sciences, Wojska Polskiego 31, 60-624 Poznan, Poland; anna.gramza@up.poznan.pl

**Keywords:** lactic acid fermentation, freeze-drying, antioxidant activity, polyphenols, probiotics, functional foods, FTIR

## Abstract

Lactic acid fermentation has emerged as a promising strategy to enhance the functional and health-promoting qualities of plant-based foods. This study evaluates the impact of lactic acid fermentation on the antioxidant capacity, microbial viability, and chemical stability of freeze-dried peaches, aiming to develop a functional food with probiotic potential. Two bacterial strains—*Fructilactobacillus fructivorans* (P_FF) and *Lactiplantibacillus plantarum* (P_LP)—were used to assess strain-dependent effects on microbial and bioactive compound profiles. Microbiological analyses included total viable count (TVC), fungal count (TFC), and total lactic acid bacteria (TCLAB). Chemical analyses comprised polyphenol, flavonoid, anthocyanin, carotenoid, sugar, and vitamin C content, as well as antioxidant activity (DPPH, ABTS, reducing power). Thermal and structural stability were examined via thermogravimetric analysis (TGA) and Fourier-transform infrared spectroscopy (FTIR). Fermentation significantly increased the counts of lactic acid bacteria, achieving 8.38 and 7.86 log CFU/g after freeze-drying, respectively. While total polyphenols slightly decreased (by 9.5% and 1.1% for *L. plantarum* and *F. fructivorans*, respectively), flavonoid content increased notably by 16.1% in *F. fructivorans*-fermented samples. Antioxidant activities, assessed by ABTS and DPPH assays, were largely maintained, although a reduction in reducing power was observed. Additionally, fermentation led to sucrose hydrolysis, resulting in higher glucose and fructose contents, and increased water content in the final products. Minor increases in total fungal counts were noted after freeze-drying but remained within acceptable limits. Overall, the combination of fermentation and freeze-drying processes preserved key antioxidant properties, enhanced microbial safety, and produced functional peach-based products with improved bioactivity and extended shelf life. These findings highlight the potential of fermented freeze-dried peaches as innovative, health-oriented alternatives to traditional fruit snacks.

## 1. Introduction

The peach (*Prunus persica*) is the third most significant fruit tree species in the Rosaceae family, thriving in temperate zones, after apples and pears. In 2022, China was the largest producer of peaches, with a harvested area of 865,144 hectares and a total production of 16,817,068 tons. This accounted for 56.1% of the world’s harvested area and 63.8% of total peach production globally. The popularity of peaches can be attributed to their sweet taste, attractive color, delightful aroma, and nutritional benefits. Peaches are rich in sugars, vitamins, proteins, organic acids, and phenolic compounds [1].

A key challenge in fruit production and distribution is its limited shelf life. Fruits are raw materials that are highly susceptible to microbiological contamination. Therefore, developing effective preservation methods is essential to ensure microbial safety and enhance sales opportunities. Traditional preservation techniques, such as refrigeration, UV sterilization, and chemical treatments, have limitations related to consumer health risks and can result in reduced nutritional value [2].

Enhancing the gastrointestinal tract with probiotics offers numerous health benefits. These benefits stem from their ability to regulate the balance of intestinal flora, manage glucose and lipid metabolism, and bolster the immune system [3]. The health benefits of fermented foods can be attributed to the competition of probiotic bacteria with harmful pathogens and the action of the biologically active peptides, short-chain fatty acids, exopolysaccharides, lactic acid, vitamins, and minerals [4,5].

Traditional food fermentation effectively manages the microbiota by carefully selecting raw materials, formulating products, and controlling fermentation processes [5]. To maintain product quality and ensure standardized production, starter cultures in food fermentation are recommended. This practice enhances quality while preserving the unique flavors characteristic of fermented products. Lactic acid bacteria affect food flavor through three key metabolic pathways. These are carbohydrate metabolism, proteolysis, catabolism, and lipolysis metabolism [6].

Lactic acid bacteria are commonly used in the food industry due to their metabolic capabilities, specifically their ability to convert carbohydrates into lactic acid during fermentation. Most microorganisms within the LAB group are classified as Generally Recognized as Safe (GRAS) and demonstrate a high tolerance to low pH environments. Morphologically, these bacteria can be found in rod and cocci shapes. They can convert plant components into lactic acid and various other products, including volatile compounds, amino acids, and active ingredients, thereby enhancing the fermented flavor [7,8].

Regarding physiology, LAB can be categorized based on their hexose fermentation pathways into two distinct groups: homofermentative and heterofermentative [9]. Homofermentative lactic acid bacteria exclusively metabolize carbohydrates into lactic acid, while heterofermentative LAB convert substrates into various products, such as lactic acid, acetic acid, and ethanol [10].

*Lactiplantibacillus plantarum* is classified within the class *Bacilli*, order *Lactobacillales*, and family Lactobacillaceae. These Gram-positive non-spore-forming rods are recognized as facultative anaerobic microorganisms [11,12]. *L. plantarum* is part of a group of homofermentative or optionally heterofermentative lactic acid bacteria (LAB), depending on the available sugars, with certain strains exhibiting probiotic properties. These microorganisms are commonly employed in producing pickled foods, which may offer various health benefits [13,14]. Additionally, *L. plantarum* can be used in the lactic fermentation of fruits, including apples and juices derived from grapes, apples, oranges, and peaches [15,16,17].

*Fructilactobacillus fructivorans* are rod-shaped bacteria with rounded ends that may occur singly, in pairs, or form various arrangements such as chains [18]. These heterofermentative lactic acid bacteria belong to the group of fructophilic lactic acid bacteria (FLAB). They are characterized by their active metabolism of D-fructose and limited growth in media containing D-glucose as the primary carbon [19,20]. These bacteria are also found in applications for producing various pickled vegetables or lactic-fermented vegetable juices [21,22].

The increasing demand for healthier diets, particularly those tailored for specific groups like vegetarians, has created new food products with functional properties. This includes products rich in bioactive compounds and those containing probiotics [23]. Probiotics can be consumed as nutraceuticals, added to food during fermentation, or taken as supplements [24,25]. A minimum concentration of viable probiotic cells must reach the intestine to ensure beneficial effects. Therefore, the suggested minimum concentration is approximately 8 log CFU/g or ml of product at the time of consumption. This recommendation accounts for the reduction in bacterial survival during gastrointestinal exposure, which should not hinder the probiotics’ functionality in the host [26]. Despite variations in daily intake recommendations from US and European agencies for health claims, a daily intake of about 6–9 log CFU of probiotics per gram or milliliter is generally considered effective [27,28,29]. However, depending on the strain, the specific dose required for health benefits may be lower. It is important to note that fermented foods should only be classified as probiotics if they meet specific criteria. Products without a defined microbiological profile should be labeled “containing live and active cultures” rather than probiotics [26,30]. Furthermore, probiotics are not limited to dairy products, for which they are most commonly known; they can also be found in vegetable and fruit products. The prebiotic effects of phenolic acids, flavonoids, and betacyanins present in plant matrices have been shown to stimulate the growth of probiotic bacteria, thereby promoting fermentation [26]. Additionally, the metabolism of probiotics within fruit matrices can enhance the bioavailability of phenolic compounds, increasing the overall functionality of the resultant products compared to the raw materials [31].

This study evaluates the impact of lactic acid fermentation on the antioxidant capacity, microbial viability, and chemical stability of freeze-dried peaches, aiming to develop a functional food with probiotic potential.

## 2. Results and Discussion

Various factors, including the chemical composition of the raw materials and the characteristics of the final product, such as taste, aroma, and texture, influence the design of new fermented products. The selection of microorganisms used in the fermentation process is contingent upon these parameters. Additionally, an important consideration is the provision of nutritional and health benefits for consumers of fermented products [29]. In this study, two types of microorganisms were selected for the fermentation of peach quarters, i.e., *L. plantarum* and *F. fructivorans*. The first synthesizes many enzymes, e.g., hydrolases, including esterases. Esterases can hydrolyze and produce esters, which have fundamental consequences for the flavor of fermented foods. Still, esterases are also involved in the bioconversion of phenolic compounds in fermented plant foods, affecting their antioxidant potential and bioavailability. On the other hand, *F. fructivorans* belongs to a recently recognized group of heterofermentative fructophilic LAB (FLAB). Their optimal growth substrate is fructose, and, in contrast to other LAB, their growth on glucose is limited. FLAB strains produce side products such as CO_2_, lactate, and acetate, while ethanol production is minimal. Notably, FLAB strains can metabolize phenolic acids, converting compounds like p-coumaric acid to phloretic acid or p-vinylphenol, and caffeic acid to dihydrocaffeic acid. These metabolites possess aromatic properties, which may positively influence the flavor of foods fermented by these bacteria [19].

### 2.1. Microbiology of Freeze-Dried Peach Samples

**Figure 1:**
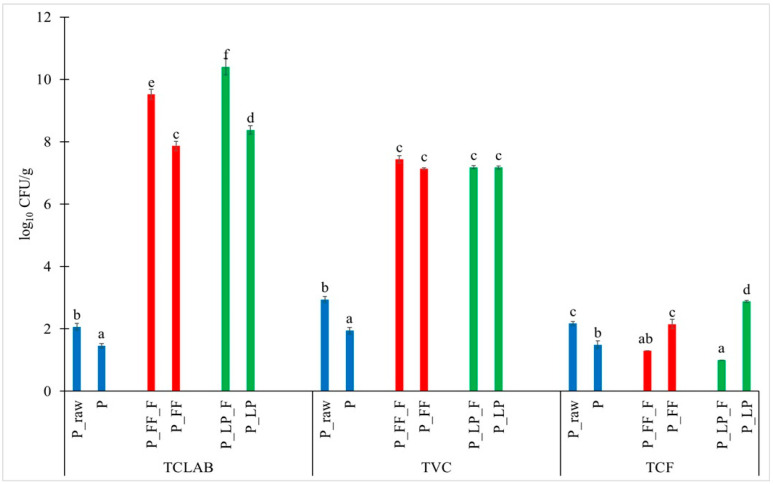
Microbiological analysis. P_raw—raw peach; P—freeze-dried unfermented peach; P_LP_F and P_FF_F—fresh peaches fermented with *L. plantarum* or *F. fructivorans*; P_LP and P_FF—freeze-dried peaches after fermentation with *L. plantarum* or *F. fructivorans*, respectively. The microbial groups, including TCLAB (total count of lactic acid bacteria), TVC (total viable count), and TFC (total fungal count), were subjected to statistical analysis using ANOVA (α = 0.05), different groups are described with lower letters a, b, c, d, e, f.

Analysis of the obtained result showed that, in fresh fruit (P_raw), the LAB count and total viable count were the lowest and obtained values of 2.07 log CFU/g and 2.94 log CFU/g, respectively (Figure 1), which may be attributed to the natural microbiota present in raw fruit materials [32]. After the freeze-drying process, values decrease to 1.46 log CFU/g and 1.95 log CFU/g, respectively (Figure 1).

Following lactic fermentation with two strains, *Lactiplantibacillus plantarum* (LP) and *Fructilactobacillus fructivorans* (FF), statistically significant differences were observed between samples. The highest TCLAB value was recorded in the P_LP_F sample, reaching 10.41 log CFU/g. Similar observations were reported by Yang et al. [33], who demonstrated that fermentation of peach pulp with *L. plantarum* resulted in a TCLAB of 9.28 log CFU/mL, significantly higher than levels observed by [34] with L. brevis, L. alimentarius, and L. pentosus. Likewise, when *L. plantarum* was used to ferment juice from various mango cultivars (“Sabre”, “Peach”, and “Tommy Atkins”), the TCLAB ranged from 8.62 to 9.31 log CFU/mL, outperforming samples fermented with L. pseudomesenteroides or mixed cultures [34]. Verón et al. [35] also reported higher viable cell counts for *L. plantarum* than F. fructosus during cactus pear juice fermentation. These findings suggest that *L. plantarum* generally exhibits superior adaptability and growth in plant-based substrates, likely due to its ability to thrive in the chemical composition of fruits such as peaches.

Microbiological analysis revealed substantial increases in bacterial counts after fermentation. Compared to the freeze-dried control (P), TCLAB increased by approximately 8 log cycles CFU/g in P_LP and 7 log cycles CFU/g in P_FF. Despite a reduction of about 2 log cycles caused by freeze-drying (e.g., from P_LP_F to P_LP), the final counts—8.38 log CFU/g for *L. plantarum* and 7.86 log CFU/g for *F. fructivorans*—remained within or above the range considered sufficient to deliver potential probiotic benefits (6–8 log CFU/g) [36]. This reduction in LAB viability is consistent with the expected effects of freeze-drying, a standard method used for the commercial preservation of probiotics, where bacterial losses are typically limited [37].

Importantly, when comparing all freeze-dried samples (P, P_LP, and P_FF), a clear enhancement in microbial counts was observed after fermentation. In the P sample (freeze-dried peach without fermentation), the TCLAB was only 1.46 log CFU/g, whereas in P_LP and P_FF, it increased to 8.38 log CFU/g and 7.86 log CFU/g, respectively. Similarly, the TVC value increased from 1.95 log CFU/g in P to 7.18 log CFU/g (P_LP) and 7.14 log CFU/g (P_FF) (Figure 1). These results confirm that lactic fermentation, followed by freeze-drying, effectively enhanced and stabilized the microbiological quality of the products compared to freeze-drying alone.

Considering the Total Viable Count (TVC) (Figure 1), a significant increase was noted post-fermentation. TVC values rose from 2.94 log CFU/g in fresh peach (P_raw) to 7.18–7.43 log CFU/g in fermented samples (P_LP_F and P_FF_F) and remained stable after freeze-drying (7.14–7.18 log CFU/g). This reflects the intense proliferation of lactic acid bacteria introduced through fermentation, which dominated the microbial community in these samples.

In contrast, the Total Fungal Count (TFC) exhibited a different trend. In fresh peaches (P_raw), TFC was 2.17 log CFU/g, but fermentation substantially reduced to approximately 1.0–1.3 log CFU/g in the fresh fermented samples. This corresponds to a 1 log cycle decrease, which can be attributed to the antifungal properties of LAB metabolites, such as organic acids and volatile compounds [38]. Such antimicrobial activity has been previously reported, for instance, in cactus pear juice fermented with *L. plantarum* and *F. fructosus* [35]. However, after freeze-drying, an increase in TFC was observed: 2.87 log CFU/g for P_LP and 2.15 log CFU/g for P_FF. Although TFC levels rose compared to fresh fermented samples, they remained relatively low and microbiologically acceptable. They were still comparable to or lower than the values recorded for the freeze-dried control (P, 1.49 log CFU/g) [19]. However, after freeze-drying, a noticeable increase in TFC was observed in both LP and FF samples.

### 2.2. Physical and Chemical Properties of Freeze-Dried Peach Samples

The analysis shows that fermenting the freeze-dried peach samples in brine with added LA bacteria caused significant changes in their physical and chemical properties. One of the changes was the increase in water content in fermented samples compared to the control (Table 1). This increase is likely due to the immersion of the dried fruit in the fermentation brine, which creates an osmotic gradient that promotes water uptake by the fruit tissues [39]. The breakdown of specific structural components during fermentation can help hold more water in the tissues. That is why the water is kept inside the samples even during freeze-drying [40]. That behavior is typical for fermentation in brine with salt addition. Water migrates into the sample tissue during that process, while minerals and sugars are transferred into the brine. In our studies, salt content in brine was relatively low; however, it can influence the migration.

A key observation involves the vitamin C content. The sample fermented with *Fructilactobacillus fructivorans* (P_FF) showed a significant drop in vitamin C levels compared to the control (Table 1). On the other hand, the sample fermented with *Lactiplantibacillus plantarum* (P_LP) kept vitamin C levels similar to the non-fermented sample. This difference suggests that the *Fructilactobacillus fructivorans* strain may act more aggressively, leading to the oxidation and degradation of vitamin C. Moreover, enzymes such as ascorbate oxidase, which can be formed during fermentation, could speed up the depletion of vitamin C [41], reducing the antioxidant ability of the P_FF sample. Lactic acid fermentation does not directly involve vitamin C levels. Nonetheless, possessing antioxidants like vitamin C could enhance preservation qualities, including minimizing oxidative damage during fermentation [42]. Similar observations were made for papaya juice [43] and guava juice [44]. Different observations were made for fermented orange juice; vitamin C content increased in this product, ensuring fermentation [45].

Significant changes were observed in the peaches’ sugar profile after fermentation. There was an increase in fructose and glucose content in both fermented samples compared to the control sample (Table 1). The sucrose content decreased by approximately 75–85% compared to the control (which contained 30.9% sucrose), indicating that enzymatic hydrolysis converted sucrose into glucose and fructose [46]. Bacterial enzymes, particularly invertase, are known to facilitate this conversion, thereby altering the sweetness and metabolic properties of the fruit [46,47]. The greater accessibility of glucose and fructose could also affect later fermentation processes, which might alter the flavor characteristics and nutritional value of the final product. Our research did not confirm the capability of FF bacterial strains to utilize fructose. *Lactobacillus plantarum* can use different sugars found in the samples, such as glucose and fructose. It has mechanisms that support the transport of these sugars and manage their usage. On the other hand, *Fructobacillus fructivorans* is focused on processing fructose and depends on external electron acceptors to use glucose, owing to its underdeveloped PTS system and unique genetic characteristics [48,49]. Although our study did not involve the fermentation of individual sugars, the observed sugar profile suggests that *F. fructivorans* (FF) utilized sugars to a lesser extent than *L. plantarum* (LP), which may reflect strain-specific metabolic activity [50,51]

### 2.3. Antioxidant Properties of Freeze-Dried Peach Samples

The results of the antioxidant activity tests using the ABTS, DPPH, and reducing power (RP) assays show how lactic acid fermentation in brine affects the antioxidant compounds in freeze-dried peach samples. The ABTS assay measures the ability to neutralize the ABTS radical cation [52]. The results indicate that the antioxidant activity of the P_FF sample was slightly lower compared to the control, as measured by the ABTS assay. This suggests that fermentation with *Fructilactobacillus fructivorans* may lower the amount of compounds that can neutralize ABTS radicals, such as polyphenols and flavonoids [53]. On the other hand, the P_LP sample has antioxidant activity similar to that of the control group. Conversely, the DPPH assay, which assesses the capacity to quench stable DPPH free radicals, reveals that the P_LP sample demonstrates lower antioxidant activity than the control and the P_FF samples. This difference suggests that fermentation with *Lactiplantibacillus plantarum* may reduce the sample’s effectiveness in neutralizing DPPH radicals. The observed reduction in activity can be attributed to structural changes in antioxidant compounds or a decrease in specific antioxidants sensitive to the DPPH assay methodology. This can be related to the fact that the highest level of LAB was also observed in LP samples compared to the FF samples.

This finding specifies that fermentation with *Lactiplantibacillus plantarum* does not significantly alter the levels of these specific antioxidant compounds. This may show a different biochemical process during fermentation and better preservation of the active compounds during fermentation. However, this was not confirmed in the tests of the active compounds; better behavior was observed for the sample fermented with *Fructilactobacillus fructivorans*.

The results of the reducing power (RP) assay indicate the capacity of antioxidant molecules to donate electrons, revealing a notable decrease in reducing power for both fermented samples in comparison to the control. This considerable drop in reducing potential (RP) implies that fermentation may have significantly reduced the overall electron-donating ability of the peaches. This decline might be attributed to the breakdown or alteration of key antioxidant compounds, such as polyphenols and flavonoids, which are known for their electron-donating properties [54].

Following the evaluation of outcomes from the ABTS and DPPH assays, it is evident that there are minor variations between the control and fermented samples. However, the significant reduction in reducing power suggests that fermenting peaches with specific bacterial strains in brine leads to notable changes in their antioxidant composition. These changes may involve a decrease in the quantity of active antioxidant compounds and alterations in their chemical structures, as confirmed by the assays of active substances detailed in point 3.4.

### 2.4. Active Substances Content in Freeze-Dried Peach Samples

Polyphenols are a group of secondary plant metabolites with a broad spectrum of biological activities. Plants synthesize them in response to adverse environmental conditions or pathogen infection [55]. Natural plant products are a rich source of polyphenols, but their bioavailability is usually limited. The process of fermentation can significantly improve their bioavailability and enhance the flavor and functional qualities of foods. During fermentation, microorganisms use nutrient compounds, including polyphenols, as metabolic substrates, leading to significant changes in the chemical composition of the final product. As a result, fermented foods rich in polyphenols have higher bioavailability of bioactive compounds and potentially higher health benefits [56].

**Figure 2:**
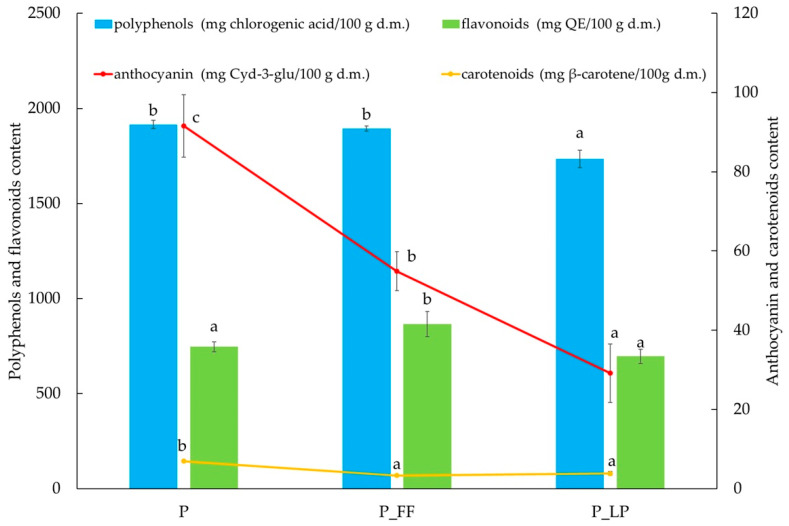
The active substances content anthocyanin, carotenoids, polyphenols, and flavonoids content; P—freeze-dried peach; P_FF—fermented with *Fructilactobacillus fructivorans* and freeze-dried peach; P_LP—fermented with *Lactiplantibacillus plantarum* and freeze-dried peach; a, b, c—results marked for series with the same lowercase letters are not statistically significantly different at α = 0.05.

The highest polyphenol content was found in the non-fermented sample (P), which contained 1916 mg/100 g d.m. The sample fermented with *Fructilactobacillus fructivorans* (P_FF) showed a slight decrease of 1.1% in TPC. In contrast, in the P_LP sample (fermented with *Lactiplantibacillus plantarum*), a reduction of 9.5% was seen (Figure 2). The observed decrease in polyphenol content in the fermented samples may be related to the action of bacterial enzymes, such as polyphenol oxidases or phenolic decarboxylases, which can degrade phenolic compounds or convert them into less reactive forms [57]. Differences between strains may be due to their different enzymatic activities, which is also supported by studies by other authors indicating that some LAB strains can metabolize polyphenols to a greater extent than others [57]. It could also be said that a greater quantity of LP bacteria in the samples may be associated with increased enzyme production.

Other results were obtained by Kwaw et al. [58] in a study on the effect of *Lactobacillus* strains on the phenolic profile of lactic acid-fermented mulberry juice. The fermented samples’ total phenolic concentration (TPC) ranged from 6.51 to 8.34 mg gallic acid/cm^3^. The non-fermented control sample had a polyphenol content of less than 6 mg gallic acid/cm^3^. In this study, there was also a significant increase in the total flavonoid content of the fermented samples compared to the control sample. These differences can be attributed to the types of sources; in our studies, we used a peach sample, not a mulberry. Moreover, in our studies, samples were cut pieces of peach, not in juice form.

A study by Zhao and Shah [59] showed that fermentation may provide an opportunity to improve the bioavailability of polyphenols. Green and black tea fermented with six strains of LAB showed an increase in many polyphenolic compounds [59].

Flavonoids are a diverse group of polyphenolic compounds commonly found in foods of plant origin (vegetables, fruits, cereals, tea, and herbal medicine). These compounds have a huge impact on human health [60,61]. Lactic fermentation of raw plant materials can contribute to the transformation of flavonoids. The right choice of LAB can increase the content of these compounds in the final product [62].

The highest flavonoid content was found in the P_FF sample (866 ± 65 mg/100 g d.m.), indicating a favorable effect of fermentation with *F. fructivorans* on the stability or biosynthesis of these compounds (Figure 2). The P_FF sample had 16.1% more flavonoids than the control (P) sample. In contrast, the P_LP sample, fermented with *L. plantarum*, showed value, with a 6.7% reduction compared to the control (P) and a 19.6% decrease relative to P_FF. *L. plantarum* may have higher enzymatic activity, leading to the degradation of some flavonoids [63]. Also *F. fructivorans* promotes the preservation or synthesis of biologically active forms due to the enzymatic degradation of complex polyphenols into simpler flavonol compounds during fermentation [64].

Fermentation of apple juice with *Lactobacillus plantarum* improved the total flavonoid content with increased free radical scavenging capacity. Before fermentation, there were 0.12 mg/mL of flavonoids in the juice, and after the fermentation process, the flavonoid content increased to 0.984 mg/mL [65].

Anthocyanins are a major group of red, purple, and blue pigments in many flowers, fruits, vegetables, and grains. They are a subclass of flavonoids and are characterized by good solubility in water. Due to their antioxidant, anti-inflammatory, and anticancer properties, anthocyanins are increasingly considered valuable dietary components that can support the prevention and treatment of selected diseases [66,67].

The anthocyanin content was highest in the unfermented sample and decreased significantly after fermentation (Figure 2). The decrease was more noticeable in the fermentation with *L. plantarum*. The degradation of anthocyanins can be related to the acidification of the environment by lactic acid bacteria and their potential oxidoreductive activity. These results indicate limited stability of anthocyanins in the fermented environment, which is consistent with observations for other lactic acid fermented plant products [68]. In a study by Hur, Lee, Kim, Choi, and Kim [68] on fermented blueberry juice, the same relationship was noted. In this study, lactic acid fermentation decreased anthocyanins, especially at a later fermentation stage. Therefore, the reduction in anthocyanin content in fermented fruit should be linked to its degradation and adsorption by lactic acid bacterial cells [63].

After fermentation with LAB *Lactobacillus plantarum*, in a study by Zhang et al. [69] on blueberry juice, the content of anthocyanins increased by 15.38% compared to fresh juice. The study analyzed the content of four different types of anthocyanins (cyanidin, petunidin chloride, pelargonidin, peonidin).

There are more than 650 types of carotenoids in nature. The benefits of their presence in the diet can be attributed to their antioxidant potential [70].

Carotenoid content was relatively low in all samples (Figure 2). Nevertheless, the control sample had the highest value (6.96 mg β-carotene/100 g d.m.), and the reduction in content in the fermented samples may be due to partial degradation of these compounds as a result of oxidation or the action of enzymes present in the fermentation system. The situation was different in a study by Escudero-López et al. [71] on fermented orange juice. The study showed a significant, high increase in content, of both flavonoids and carotenoids, after fermentation compared to samples that were not fermented.

The study by Xu et al. [72] showed that fermentation by *Lactobacillus gasseri* enables the production of carrot juices with probiotic bacteria and carotenoids. However, the researchers did not determine the exact carotenoid content of the resulting products.

### 2.5. Thermal Stability and Chemical Structure of Freeze-Dried Peach Samples (TGA and FTIR Analysis)

The freeze-dried fermented peach molecular composition and particle structure were investigated using Fourier transform infrared spectroscopy (FT-IR). This technique assesses the vibrational energy between atoms within the samples. FT-IR can distinguish between freeze-dried and fermented peach samples by identifying specific spectral variations that indicate different chemical compositions and structural changes resulting from the drying process [73].

**Figure 3:**
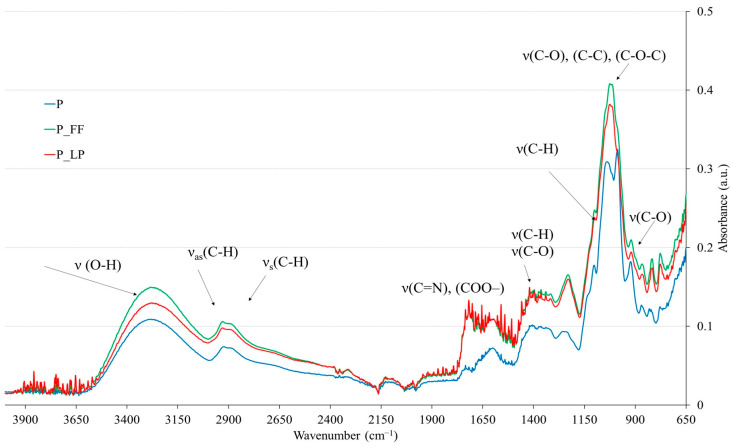
FT-IR spectrum analysis for peach samples. P—freeze-dried peach; P_FF—peach fermented with *F. fructivorans* then freeze-dried; P_LP—peach fermented with *L. plantarum* then freeze-dried.

The FTIR spectra (Figure 3) show samples fermented with different LAB strains alongside a control sample, illustrating the relationship between absorbance intensity and energy in wavenumbers (cm^−1^). Similar functional groups across the samples indicate that uniform fermentation products are derived. Analyzing the FTIR spectra of the peach samples (P, P_FF, and P_LP) reveals some differences in their chemical composition, primarily attributed to the fermentation process.

Within the 3600–3000 cm^−1^ range, broadband corresponding to O-H stretching vibrations, it can be linked to hydroxyl groups in water, sugars, and those present in polyphenols structure [74,75]. The variations in intensity among the samples suggest alterations in hydroxyl-containing compounds resulting from fermentation, mainly in the regions of 1600–900 cm^−1^.

Between 3000 and 2800 cm^−1^, the spectra show absorption bands related to asymmetric (νas(C-H)) and symmetric (νs(C-H)) stretching vibrations of C-H bonds. Similar observations were made for apples. In their research, the peaks at 2930 cm^−1^, 2885 cm^−1^, and 2850 cm^−1^ are linked to the asymmetric stretch vibration of aliphatic CH_3_ and CH_2_ groups, and symmetrical stretch vibration of aliphatic CH_3_ groups, respectively [76]. In our samples, the highest peak in these bands can indicate modifications in the composition of these macromolecules following fermentation. Additionally, in the 2250–2000 cm^−1^ range, a characteristic band corresponding to C≡C stretching vibrations and COO^−^ groups is present, suggesting the formation of organic compounds such as amino acids, or fermentation metabolites [74,77].

In the 1750–1500 cm^−1^ region, a distinct band appears around 1650 cm^−1^, corresponding to C=O stretching vibrations typically associated with esters, carboxylic acids, polyphenols, and pectins [78]. The variations in intensity and position among the samples indicate that fermentation influenced these functional groups’ presence and structural arrangement. Furthermore, the range between 1500 and 900 cm^−1^ exhibits strong absorption bands assigned to C-O, C-C, and C-O-C stretching vibrations, characteristic of carbohydrates, cellulose, hemicelluloses, and pectins [78]. The observed differences between the spectra of fermented and non-fermented samples suggest that bacterial fermentation led to polysaccharide degradation or structural modifications in sugars [79,80].

Weaker sucrose bands (~990–1000 cm^−1^) and enhanced broad OH signals (~3200–3600 cm^−1^) in fermented samples agree with the hydrolysis of sucrose into glucose/fructose and loss of phenolics. This matches the chemical analyses (Table 1) and reduced RP values. The decrease in RP values (Table 1) in both fermented samples indicates the degradation of reducing antioxidants like polyphenols, with a more significant reduction observed in the P_LP sample (Figure 2). In the FTIR spectra, a diminished intensity of bands is associated with phenolic groups, specifically around 3200–3600 cm^−1^ for -OH and 1500–1600 cm^−1^ for the aromatic ring. The FTIR results demonstrate that fermentation altered the chemical composition of the freeze-dried peach samples.

Fourier transform infrared spectroscopy (FT-IR) was used to compare the molecular composition and particle structure of freeze-dried fermented peach samples. The spectra show typical absorption bands related to O-H, C-H, C=O, and polysaccharide functional groups. Some differences between fermented and non-fermented samples were observed, reflecting changes in chemical composition due to fermentation, such as modifications in hydroxyl groups, carbohydrates, and phenolic compounds. However, given the complex nature of the peach matrix, these spectra should be interpreted cautiously and mainly as comparative profiles rather than detailed molecular identification.

Similar observations with regions were observed for dried apples [76,81], fermented pears [82], and fruits and vegetable wastes [83,84].

### 2.6. TGA

**Figure 4:**
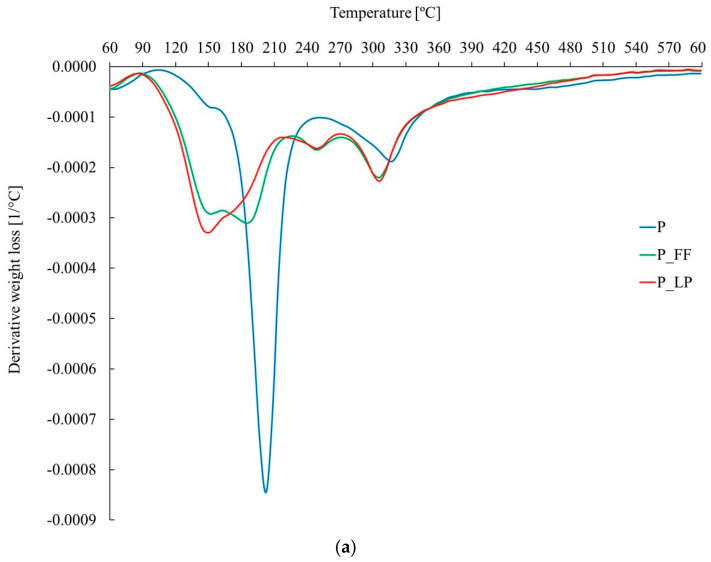

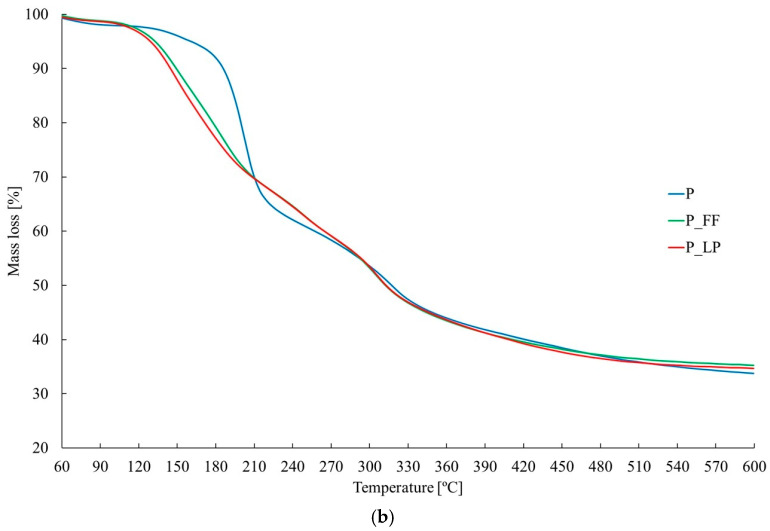
Thermogravimetric analysis (TGA) curves for peach samples, control (P), and fermented (P_FF; P_LP): (**a**) derivative weight loss, (**b**) mass loss; P—freeze-dried peach sample; P_LP—fermented with *Lactiplantibacillus plantarum* and freeze-dried peach samples; P_FF—fermented with *Fructilactobacillus fructivorans* and freeze-dried peach sample.

Thermogravimetric analysis (TGA) was used to evaluate the thermal stability of the freeze-dried fermented peach samples and the integrity of cell wall polymers [85]. The TGA curves revealed four distinct decomposition stages corresponding to moisture loss, degradation of low-molecular-weight compounds, polysaccharide breakdown, and stable residue decomposition (Figure 4). Fermentation influenced mass loss at different stages: P_FF lost less moisture, while P_LP showed reduced loss of low-molecular-weight compounds. Differences in decomposition temperatures suggest that fermentation alters the thermal behavior and chemical composition of the samples without significantly affecting their overall thermal stability (Table 2). Both fermented and non-fermented samples exhibit four distinct phases, each linked to specific chemical reactions [86].

All samples showed four decomposition stages (Figure 4). The first stage (30–95 °C) involves moisture evaporation and volatile compound release [87]. In the second stage (90–270 °C), the breakdown of low-molecular-weight compounds leads to the highest mass loss. The third stage (220–380 °C) corresponds to polysaccharide degradation [88,89]. The fourth stage (380–602 °C) involves stable organic residue degradation.

Fermentation changed mass loss at various stages: P_FF lost less moisture (<95 °C), whereas P_LP lost fewer low-MW compounds (90–270 °C). In the polysaccharide region (220–380 °C), P_LP lost more mass (+33.7%) and P_FF less (–12.8%) relative to P (Table 2). Overall, thermal stability (total mass loss) was similar among samples, but P_FF decomposed at lower peak temperatures (149–183 °C) than P (199 °C), indicating more thermolabile compounds from fermentation. All samples behaved alike above 380 °C. The total mass loss among samples remains similar, suggesting that fermentation does not significantly alter overall thermal stability but affects decomposition stages (Table 2).

Decomposition temperatures vary, with the control sample (P) showing the highest temperature in the second stage (199.7 °C), suggesting higher stability. P_FF decomposes earlier (149.3 °C), indicating fermentation accelerates degradation, while P_LP has an intermediate profile (Table 2). A completely different thermal profile was observed in the samples after fermentation. In sample P_FF the main thermal transformations already took place at 149.3 °C and 182.8 °C, indicating the presence of more volatile and thermolabile compounds that are fermentation products. Additional peaks at 246.3 °C and 302.1 °C suggest a complex sample composition and possible secondary transformations. For sample P_LP, no temperature in the range of 180 was observed (Table 2 and Figure 4a,b)

The mass loss curves indicate that sample P decomposes rapidly between 90 °C and 250 °C, whereas the fermented samples (P_FF and P_LP) exhibit a more gradual decline, reflecting changes induced by fermentation (Figure 4b). At temperatures above 300 °C, all samples demonstrate similar decay behavior related to the stable sludge.

The DTG curves reveal distinct peaks corresponding to different decomposition phases (Figure 4a). Sample P shows a sharp peak around 200 °C, which indicates rapid decomposition, while the fermented samples P_FF and P_LP display broader peaks, suggesting a more gradual decomposition process. Additionally, the second peak, occurring in the 310–350 °C range, corresponds to polysaccharide degradation, with sample P_LP showing higher retention than sample P_FF.

The observed differences in thermal behavior between fermented and non-fermented samples may suggest that lactic fermentation causes structural modifications to the peach matrix. These modifications may include the partial decomposition or transformation of components that decompose easily, such as simple sugars and volatile compounds. This affects thermal stability at lower temperature ranges. While specific classes of compounds cannot be identified with certainty from TGA data, shifts in decomposition profiles provide indirect evidence of changes in the physicochemical composition. In contrast, the presence of similarities in the high temperature range (>380 °C) for all samples confirms that fermentation does not significantly affect the most thermally stable components, such as residual carbonaceous material.

Differences in thermal behavior between the two fermentation strains (P_FF and P_LP) suggest that microbial metabolism during fermentation can selectively interact with different macromolecules in fruit tissue, thereby affecting overall decomposition dynamics. These results may be useful for optimizing fermentation processes to improve the thermal stability or drying efficiency of fruit products.

## 3. Materials and Methods

### 3.1. Materials

Peaches (*Prunus persica* L.) purchased from a local store in Warsaw were used for the study. The fruits were selected for comparable size, color, and ripeness. Before further processing, the peaches were stored in the refrigerator (4 °C, relative humidity 95%) for one day. They were then washed under running water and immersed in a 0.05% sodium hypochlorite solution for 2 min for surface disinfection [90]. After this process, the fruits were dried on blotting paper, cut into eight equal parts, and the seeds were removed. The prepared pieces were placed in sterile 500 mL glass jars, dispensing 200 g of fruit per jar. The peaches were then poured over 200 mL of a 0.5% sodium chloride solution.

The chemical reagents used for analysis were of analytical grade.

### 3.2. Fermentation

Fermentation was carried out in two variants, using strains of *Lactiplantibacillus plantarum* ATCC 4080 (LP) obtained from the American Type Culture Collection (ATCC, Manassas, VA, USA), and *Fructilactobacillus fructivorans* DSM 20203 (FF) from Leibniz Institute DSMZ-German Collection of Microorganisms and Cell Cultures GmbH (DSMZ, Braunschweig, Germany), which were stored frozen. The inoculum corresponded to cultures of selected strains at approximately 1 × 10^8^ CFU/mL. The inoculum was prepared in a 0.85% NaCl solution using a Densimat (BioMérieux, Craponne, France). These conditions ensured consistent inoculum distribution and facilitated effective lactic acid fermentation. Due to our previous pre-tests and studies on other raw materials, the fermentation time was carefully selected. The fermentation temperature of 25 °C was chosen as this was found to be optimal for the growth of the tested microorganisms, ensuring effective fermentation without significant loss of bioactive compounds. The fermentation process was carried out over six days at a constant temperature of 25 °C in an incubator (BD-S115, Binder, Tuttlingen, Germany). The fermented samples P_LP and P_FF were prepared using the same method. First, the cut peach samples were placed into glass jars. Next, a solution of water containing 0.5% NaCl was added. Then, a 10% *v*/*v* inoculum was added to the jars containing the peach samples. Finally, the jars were sealed to prevent air from entering.

### 3.3. Freeze-Drying

After fermentation, the fruits were drained and placed on metal trays and frozen in a blast freezer (HCM 51.20, Irinox, Treviso, Italy) at −40 °C for 10 h. After the freezing process, the samples were freeze-dried. The temperature of the freeze-dryer shelf (ALPHA 1–4, Martin Christ Gefriertrocknungsanlagen GmbH, Osterode am Harz, Germany) was set at 25 °C, and the chamber pressure was 0.630 mbar. The temperature of the condenser was kept constant at −55 °C throughout the process. Drying was carried out until a constant weight of material was obtained.

The control sample in the study was freeze-dried unfermented peaches, which underwent the same stages of preparation, freezing, and freeze-drying, with the fermentation process omitted. Products for further analysis were stored in polyethylene bags (PET12/Al8/PE100), a barrier to light and moisture at 24 °C.

### 3.4. Microbiological Tests

The pour plate method was used to determine the total viable count (TVC), the total count of fungi (TCF), and the count of lactic acid bacteria (TCLAB). Under sterile conditions, 10 g of peach was mixed with 90 mL of sterile saline (0.85% NaCl) and then homogenized for 1 min (Stomacher 400 Circulator, Seward, UK). The homogenate was then serially diluted. These dilutions were poured into plates with agar counts: PCA (plate count agar) for TVC, DRBC (dichloran rose bengal chloramphenicol agar) to determine TCF, and MRS (de Man, Rogosa and Sharpe) for TCLAB. PCA and MRS plates were incubated at 30 ± 2 °C, and DRBC plates at 25 ± 2 °C, each for 48–72 h (Binder, Tuttlingen, Germany). The count of grown microbial colonies was counted (ProtoCOL 3—Automatic colony counting and zone measuring, Synbiosis, Frederick, MD, USA) and then recorded as log CFU per g. The limit of detection was 1.0 log CFU/g [91]. Samples were analyzed in triplicate. All mediums and reagents used in the research were purchased from Biomaxima (Lublin, Poland).

### 3.5. Water Content

For water content determination, the gravimetric method was used according to the methodology from Karwacka et al. [80]. A known mass of each sample was weighed into vessels and placed in a drying oven at 70 °C for 24 h. After drying, the samples were weighed again, and the water content was calculated by determining the percentage of mass lost relative to the initial weight.

### 3.6. Chemical Compounds Analysis

#### 3.6.1. The Total Sugar Content

Total sugar content was determined using high-performance liquid chromatography (HPLC) equipped with an infrared (IR) detector (Waters, Milford, MA, USA). For each analysis, 300 mg of ground sample was extracted in 10 mL of Milli-Q water (resistivity: 18.2 MΩ cm) at 80 °C for 4 h, 1200 rpm on a Multi Reax shaker. After extraction, the mixture was centrifuged (4350 rpm, 2 min). The supernatant was then filtered through an 0.22 µm Acrodisc PSF GHP syringe filter (Pall Life Sciences, New York, NY, USA). Chromatographic separation was performed on a Sugar-Pak I cation-exchange column (6.5 mm × 300 mm, 10 µm; Waters, Milford, CT, USA) at 90 °C, with a Sugar-Pak Guard-Pak (10 µm) guard column. Each run used a 10 µL injection volume with isocratic elution using Milli-Q water as the mobile phase at a flow rate of 0.6 mL min^−1^ for a total run time of 20 min [92]. Quantification was conducted using calibration curves (0–5 000 µg/mL) for standard solutions of sucrose, D-(+)-glucose, and D-(−)-fructose (Sigma-Aldrich, Steinheim, Germany). All measurements were carried out in triplicate.

#### 3.6.2. Vitamin C

Vitamin C was quantified using an ACQUITY UPLC H-Class System (Waters) system equipped with a photodiode array detector [93]. To minimize vitamin C degradation, sample extraction was performed immediately before analysis. A total of 50 mg of finely ground sample was weighed and homogenized with 10 mL of a chilled extraction solution consisting of 3% metaphosphoric acid and 8% acetic acid. The mixture was vortexed for 10 min and centrifuged at 6000 rpm for 5 min at 4 °C. A dithiothreitol (DTT) solution (1 g L^−1^) was employed as a reducing agent to enable the quantification of vitamin C (sum of ascorbic acid and dehydroascorbic acid). The supernatant was diluted twice with the DTT solution and incubated for 1 h at 4 °C before further analysis. All procedures were conducted under reduced light exposure to prevent photodegradation. The solution was filtered through a 0.22 µm (PSF GHP Acrodisc filter (Pall Gelman, Ann Arbor, MI, USA). An aliquot of 5 µL was injected into a WATERS Acquity UPLC HSS T3 column (2.1 × 100 mm, 1.8 µm; Waters, Ireland) for chromatographic separation. The mobile phase was delivered at a flow rate of 0.25 mL min^−1^, the column oven was maintained at 25 °C, and the autosampler was kept at 4 °C. Detection was performed at a wavelength of 245 nm. Vitamin C concentration was quantified using an external calibration curve prepared using L (-) ascorbic acid standards (Sigma-Aldrich, St. Louis, MO, USA) in the 0.005–0.1 mg mL^−1^ concentration range. All measurements were conducted in duplicate.

#### 3.6.3. Antioxidant Properties

##### Extract Preparation

The extract was prepared similarly to analyze polyphenol content and antioxidant activity. The material was ground using an analytical mill (IKA-A11, Merck, Darmstadt, Germany). A total of 300 mg of the dried material was extracted with 10 mL of extraction reagent (80% ethanol solution with 15% 0.1 M hydrochloric acid). The extraction was carried out using an orbital shaker for 12 h under limited light exposure at a temperature of 18 °C. After the process, the solution was centrifuged and transferred to a PCR tube. Until analysis, the extract was stored at −20 °C.

##### Total Phenolic Content (TPC)

The total phenolic content in peach was determined using a spectrophotometric method based on a color reaction with the Folin–Ciocalteu reagent [94]. The extracts were diluted twice with distilled water, and the reactions were carried out in 96-well plates. A 5-fold diluted Folin–Ciocalteu reagent (40 µL) was mixed with 10 µL of the extract, followed by the addition of 250 µL of a 7% sodium carbonate solution after 3 min. The mixture was incubated for 60 min at room temperature, protected from light. Absorbance was measured at 750 nm using a Multiskan Sky plate reader (Thermo Electron Co., Ltd., Waltham, MA, USA). A blank sample, where the extraction reagent replaced the extract, was also measured. Each extract was analyzed in duplicate. A calibration curve was constructed using chlorogenic acid in concentrations ranging from 0 to 100 µg/mL to quantify the polyphenol content. The results were expressed as milligrams of chlorogenic acid per 100 g of dry matter.

##### Antioxidant Activity (ABTS and DPPH)

The ability to neutralize ABTS and DPPH radicals was assessed following previously described methods [95]. Radical solutions were prepared 24 h before the analysis by dissolving 25 mg of 2,2-diphenyl-1-picrylhydrazyl (DPPH) in 99% methanol and diluting to 100 mL, and by dissolving 38.4 mg of 2,2′-azino-bis(3-ethylbenzothiazoline-6-sulfonic acid) (ABTS) in 10 mL of distilled water, with the addition of 6.6 mg of potassium persulfate. Just before the analysis, the stock solutions were diluted with 80% ethanol to adjust the absorbance within the range of 0.68–0.72. To determine antioxidant activity, 10 μL of the extracts was added to a 96-well plate, mixed with 250 μL of the respective radical solution. The plate was shaken and incubated in the dark for 2 h. After incubation, the absorbance of the samples was measured using a Multiskan Sky plate reader (Thermo Electron Co., Ltd., Waltham, MA, USA) at 734 nm for the ABTS assay and 515 nm for the DPPH assay. The antioxidant activity was calculated and expressed as Trolox equivalents in mg Trolox per gram of sample.

##### RP (Reducing Power)

To evaluate the reducing power of iron (III) ions by the sample, 25 μL of the extract, 50 μL of a 1% potassium ferricyanide solution, and 75 μL 200 mM phosphate buffer (pH 6.6) were added to a well. The mixture was then incubated at 50 °C. After 20 min, 50 μL of 10% trichloroacetic acid (TCA) was introduced. Following this, 100 μL of the reaction mixture was transferred to a clean well, where 100 μL of distilled water and 20 μL of a 0.1% iron (III) chloride solution were added and mixed. After 10 min, the absorbance was measured at 700 nm against a blank [96]. The reducing power of iron (III) ions was quantified as mg of Trolox/g d.m.

##### Flavonoids

The total flavonoid content was assessed using aluminum (III) chloride, as described in reference [97]. In brief, 20 µL of the sample extract was combined with 80 µL of distilled water, followed by the addition of 10 µL of 5% (*w*/*v*) sodium nitrite. After 5 min, 10 µL of a 10% (*w*/*v*) aluminum chloride solution was introduced and mixed. Then, after 6 min, 40 µL of a 1 M sodium hydroxide solution was added and thoroughly mixed. Following a 20 min incubation period, the absorbance was recorded at 510 nm. The flavonoid concentration was determined using a standard curve prepared with quercetin across a concentration range of 0–500 µg/mL. Each measurement was performed in duplicate.

### 3.7. Pigments

#### 3.7.1. Total Carotenoids Content (*TCC*)

The total carotenoid content was assessed following a modified protocol based on the work by Tabaszewska et al. [98]. A total of 0.2 g of the sample was homogenized with 15 mL of distilled water to eliminate interfering substances such as proteins. Subsequently, 1 mL each of Carrez Solution I and Carrez Solution II (Carl Roth GmbH, Karlsruhe, Germany) was added to the mixture. After thorough mixing, the solution underwent centrifugation to separate the precipitate. The resulting precipitate was subjected to two sequential extractions using 20 mL portions of acetone, each for 4 min, employing an orbital shaker. Post-extraction, the mixtures were centrifuged at 1000× *g* for 7 min using a laboratory centrifuge (Megastar 600R, VWR). The combined supernatants were transferred to a separatory funnel containing 50 mL of petroleum ether and 10 mL of distilled water. The mixture was allowed to separate, and the upper organic phase was collected. This phase was then dried over 1.5 g of anhydrous sodium sulfate to remove residual moisture.

The absorbance of the purified extract was measured at 450 nm using a spectrophotometer (Spectronic 200; Thermo Fisher Scientific Inc., Waltham, MA, USA). All measurements were performed in triplicate to ensure accuracy.

The total carotenoid content (*TCC*) was calculated using the following equation:

*TCC* = *ABS* × 105 × *m*_2_/4.0 × *m*_1_ × *dm* (1)

where:

*ABS* is the absorbance at 450 nm;
*m*_2_ is the total weight of the extract (g);
4.0 is the extinction coefficient of β-carotene in petroleum ether;
*m*_1_ is the initial sample weight (g);
and *dm* represents the dry matter content of the sample.

#### 3.7.2. Anthocyanin

The total anthocyanin content was quantitatively determined using the differential pH method [99,100]. For this analysis, two buffers were prepared: one with pH 1 (1.86 g KCl in 1 L of water, adjusted to pH 1 using concentrated HCl) and another with pH 4.5 (54.43 g of CH_3_CO_2_Na · 3H_2_O in 1 L of water, adjusted to pH 4.5 with concentrated HCl). The reactions were performed on a 96-well plate. To each well, 30 µL of the extract solution and 135 µL of buffer were added and mixed. After incubating for 20 min at 25 °C, the absorbance was measured at 510 and 700 nm using a Multiskan Sky plate reader (Thermo Electron Co., Ltd., Waltham, MA, USA). A reagent blank, consisting of 30 µL of the extraction solvent and 135 µL of buffer, was also measured simultaneously. The result was expressed as mg Cyd-3-glu/100 g d.m. Each extract was analyzed in duplicate.

The total anthocyanin content (TAC) was calculated using the following equation:

TAC = (ABS · MW · DF · 1000)/(ϵ · L)(2)

where:

ABS = (ABS_510_ − A_70BS0_) pH1 − (ABS_510_ − ABS_700_) pH4.5;
MW = molecular weight of cyanidin-3-glucoside (449.2 g/mol);
DF = dilution factor of the sample;
ϵ = molar absorption coefficient of cyanidin-3-glucoside (26,900 L/mol·cm);
L = optical path length of the solution in the well (0.173 cm).

### 3.8. Chemical Compounds—Fourier Infrared Spectroscopy (FT-IR)

The infrared absorption spectra of freeze-dried samples were recorded using a Cary 630 FTIR spectrometer (Agilent Technologies Inc., Santa Clara, CA, USA) equipped with a diamond attenuated total reflectance (ATR) accessory [101]. Spectral data were collected over the mid-infrared range of 650–4000 cm^−1^ at a resolution of 4 cm^−1^, averaging 32 scans per sample to enhance the signal-to-noise ratio. Prior to each measurement, a background spectrum was acquired under identical conditions to account for atmospheric and instrumental contributions. Data acquisition and processing were conducted using Agilent’s MicroLab PC 5.7 software, which facilitates intuitive operation and reliable spectral interpretation

### 3.9. Thermal Analysis (TGA)

Thermal characteristics of the samples were analyzed using a thermogravimetric analyzer (TGA/DSC 3+, Mettler Toledo, Greifensee, Switzerland) to monitor weight changes during controlled heating. Approximately 5–7 mg of freeze-dried peach samples was placed in alumina crucibles and subjected to a temperature increase from 30 °C to 600 °C at a constant rate of 5 °C/min under a nitrogen atmosphere flowing at 50 mL/min [102]. The resulting thermogravimetric (TG) and derivative thermogravimetric (DTG) curves were recorded and analyzed to identify decomposition stages and corresponding peak transition temperatures. Data processing was performed using STARe Evaluation Software (version 16.10, Mettler Toledo), facilitating precise interpretation of thermal degradation profiles. Each measurement was conducted in duplicate to ensure the reproducibility and reliability of the results.

### 3.10. Statistical Treatment

Data in the article are presented as a means ± standard deviation. The collected data were statistically analyzed using one-way analysis of variance (ANOVA) to determine the significance of differences between mean values of the examined parameters. Tukey’s post hoc test was applied to pairwise comparisons and identify statistically distinct groups when a significant effect was observed. A significance level of α = 0.05 was used for all tests. Statistical analyses were conducted using Statistica software, version 13.1 (TIBCO Software Inc., Palo Alto, CA, USA).

## 4. Conclusions

Lactic acid fermentation, followed by freeze-drying, represents a viable methodology for the production of shelf-stable fruit snacks that exhibit enhanced microbial safety and functional properties. The current study investigated peaches that underwent fermentation with *Lactiplantibacillus plantarum* and *Fructilactobacillus fructivorans*, resulting in probiotic-relevant levels of bacterial activity post-freeze-drying, quantified at 8.38 log CFU/g and 7.86 log CFU/g, respectively. The fermentation process facilitated the hydrolysis of sucrose into glucose and fructose, led to an increase in water content, and preserved antioxidant activity as measured by ABTS and DPPH assays, despite a concomitant reduction in reducing power. Although there was a nominal decrease in total polyphenol content, a notable increase in flavonoid levels was documented in the *F. fructivorans* sample. Following freeze-drying, fungal counts exhibited a slight increase, yet these remained within acceptable regulatory limits.

Further examination through dedicated physical and sensory evaluations, elaborated upon in a complementary article, revealed significant physicochemical and structural modifications, including a 6–7% reduction in dry matter. Microscopic and microtomographic analyses indicated high porosity and a loosening of cell walls, particularly in samples treated with *F. fructivorans*, which enhanced water vapor permeability and moisture absorption. The fermentation process caused a reduction in brightness and a shift in total color difference to higher values, potentially influencing consumer perception. Sensory analysis indicated that the fermented samples received lower scores on attributes such as sweetness, crunchiness, and overall acceptability in comparison to the control sample.

Both tests showed that fermentation can improve microbial quality and enrich the profiles of bioactive compounds, and this process may also adversely affect texture and flavor. It is posited that process optimization—by adjusting fermentation conditions or modifying the sugar content in the brine—could support lactic acid bacteria (LAB) activity while preserving the sensory characteristics that are deemed desirable. However, an increase in sugar concentration post-drying, aimed at enhancing flavor, risks compromising textural integrity due to alterations in porosity. It is essential to achieve a careful balance between health benefits and sensory attributes.

## Figures and Tables

**Table 1:** Selected physical and chemical results for freeze-dried peach samples.

Sample Code	P	P_FF	P_LP
Water content (%)	3.1 ± 0.1 a	10.3 ± 0.0 c	9.1 ± 0.3 b
Vitamin C (mg/100 g d.m.)	109.1 ± 3.5 b	81.6 ± 5.5 a	102.3 ± 8.7 b
Fructose (%)	6.2 ± 0.1 a	10.6 ± 0.6 b	10.2 ± 0.3 b
Glucose (%)	5.2 ± 0.3 a	10.9 ± 0.2 c	9.1 ± 0.3 b
Sucrose (%)	30.9 ± 0.3 c	7.6 ± 0.2 b	4.6 ± 0.0 a
ABTS (mg TE/g d.m.)	3.5 ± 0.1 b	3.1 ± 0.2 a	3.4 ± 0.0 ab
DPPH (mg TE/g d.m.)	47.0 ± 2.5 b	46.3 ± 2.3 ab	40.1 ± 2.8 a
RP (mg TE/g d.m.)	18.2 ± 0.5 b	12.7 ± 1.0 a	12.9 ± 1.5 a

Values are mean ± SD (*n* = 3); values in the same row with different letters are significantly different (Tukey’s test, *p* < 0.05). d.m.—Dry matter (d.m.).

**Table 2:** Thermogravimetric information for tested peach samples (TGA results).

Sample Code	Step 1		Step 2		Step 3		Step 4		Sum [%]	Decomposition Temperature [°C]			
	Temp. Range (°C)	Mass Loss (%)	Temp. Range (°C)	Mass Loss (%)	Temp. Range (°C)	Mass Loss (%)	Temp. Range (°C)	Mass Loss (%)		2			3
P	30–95	2.0	95–245	36.0	245–380	19.6	380–600	8.7	66.2	199.7			314.6
P_FF	30–95	1.2	95–270	39.7	270–380	17.1	380–601	6.7	64.7	149.3	182.8	246.3	302.1
P_LP	30–90	1.4	90–220	30.7	220–380	26.2	380–602	7.3	65.6	146.9		246.3	304.0

P—freeze-dried peach; P_FF—peach fermented with *F. fructivorans* then freeze-dried; P_LP—peach fermented with *L. plantarum* then freeze-dried. Cells marked in black indicate no phase change detected within that temperature range.

## Data Availability

The authors confirm that all data supporting the findings of this study are available within the article.
